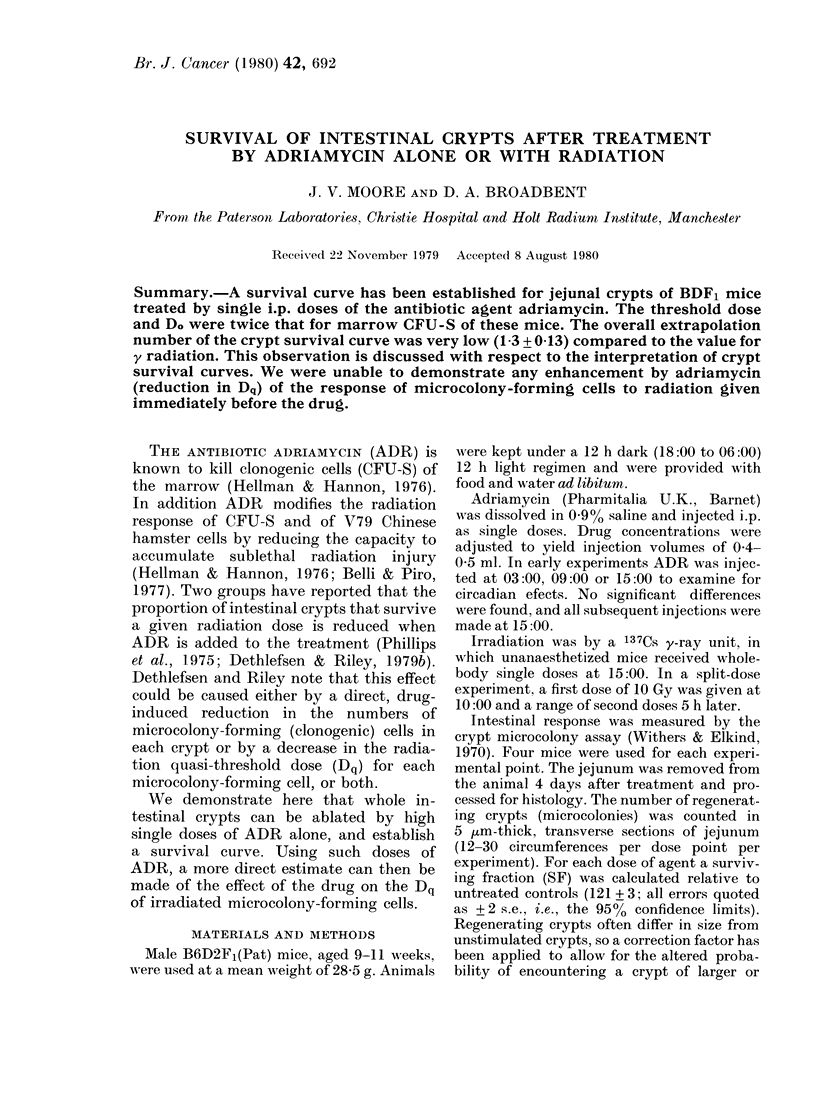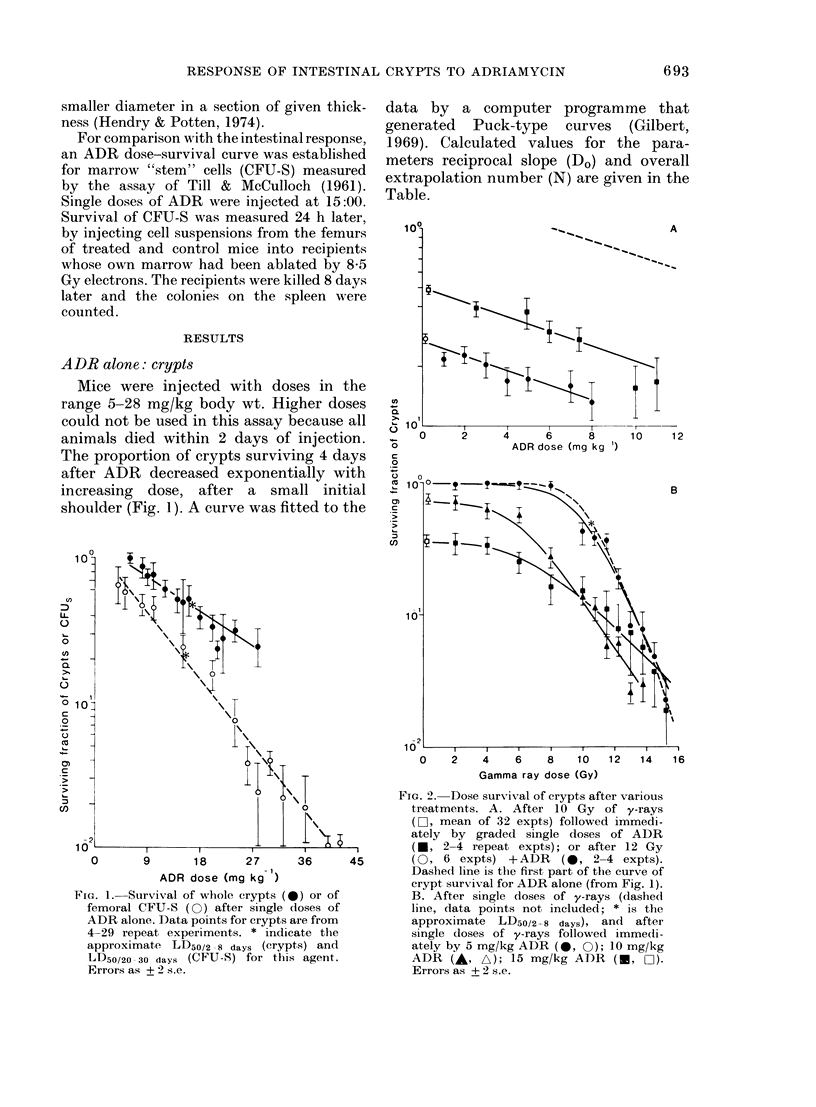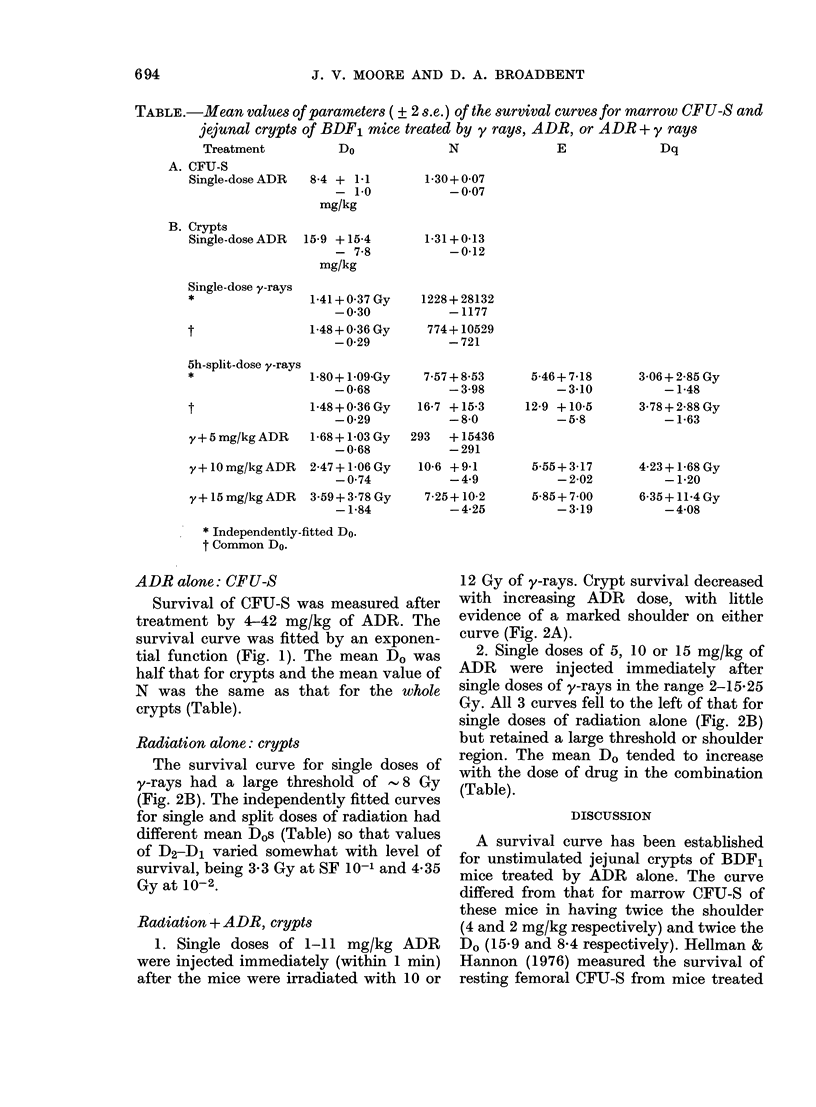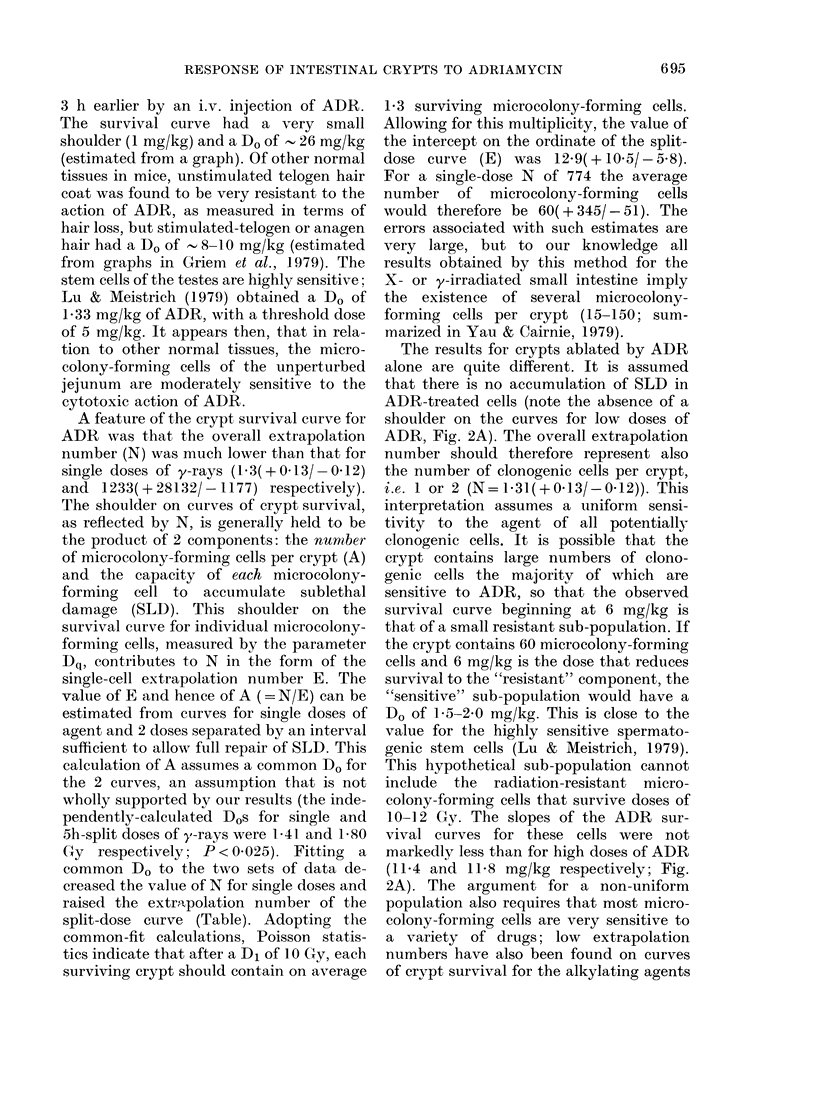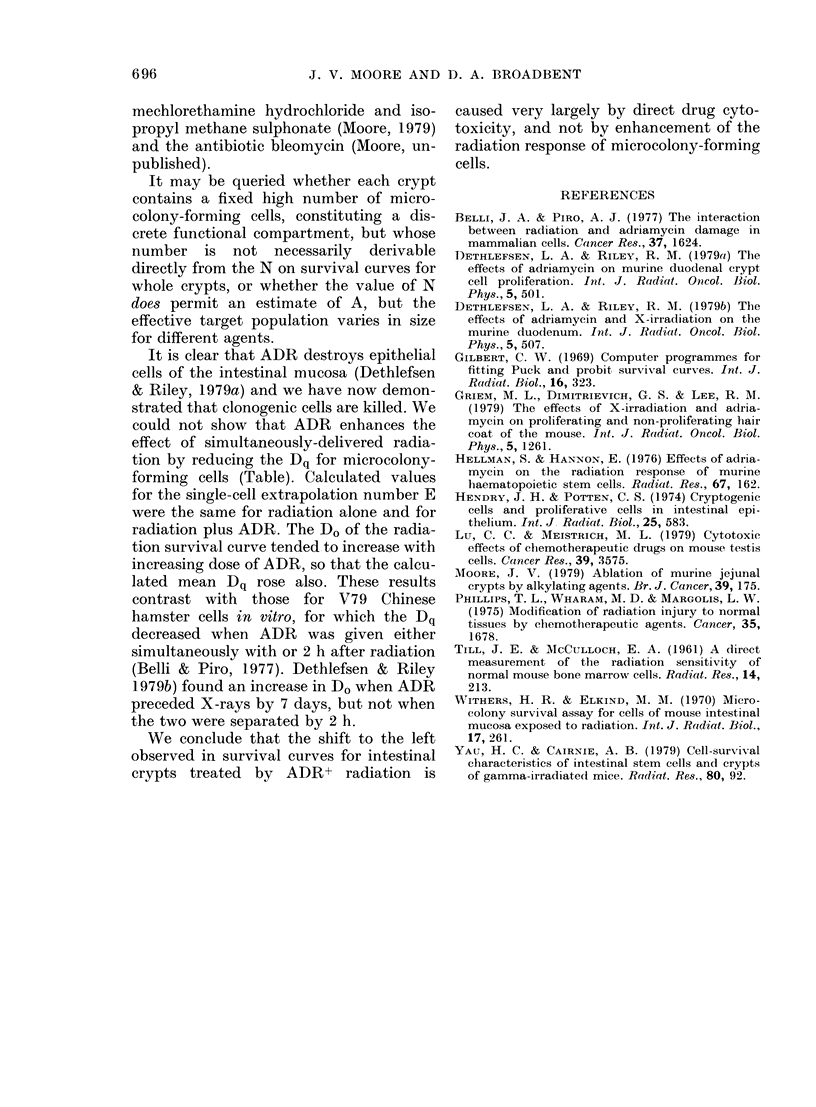# Survival of intestinal crypts after treatment by adriamycin alone or with radiation.

**DOI:** 10.1038/bjc.1980.303

**Published:** 1980-11

**Authors:** J. V. Moore, D. A. Broadbent

## Abstract

A survival curve has been established for jejunal crypts of BDF1 mice treated by single i.p. doses of the antibiotic agent adriamycin. The threshold dose and Do were twice that for marrow CFU-S of these mice. The overall extrapolation number of the crypt survival curve was very low (1.3 +/- 0.13) compared to the value for gamma radiation. This observation is discussed with respect to the interpretation of crypt survival curves. We were unable to demonstrate any enhancement by adriamycin (reduction in Dq) of the response of microcolony-forming cells to radiation given immediately before the drug.


*Br. J. Cancer* (1980) **42**, 692

### SURVIVAL OF INTESTINAL CRYPTS AFTER TREATMENT BY ADRIAMYCIN ALONE OR WITH RADIATION

J. V. MOORE AND D. A. BROADBENT

*From the Paterson Laboratories, Christie Hospital and Holt Radium Institute, Manchester*

Received 22 November 1979 Accepted 8 August 1980

**Summary.—A survival curve has been established for jejunal crypts of $BDF_1$ mice treated by single i.p. doses of the antibiotic agent adriamycin. The threshold dose and $D_o$ were twice that for marrow CFU-S of these mice. The overall extrapolation number of the crypt survival curve was very low ($1{\cdot}3 \pm 0{\cdot}13$) compared to the value for $\gamma$ radiation. This observation is discussed with respect to the interpretation of crypt survival curves. We were unable to demonstrate any enhancement by adriamycin (reduction in $D_q$) of the response of microcolony-forming cells to radiation given immediately before the drug.**

THE ANTIBIOTIC ADRIAMYCIN (ADR) is known to kill clonogenic cells (CFU-S) of the marrow (Hellman & Hannon, 1976). In addition ADR modifies the radiation response of CFU-S and of V79 Chinese hamster cells by reducing the capacity to accumulate sublethal radiation injury (Hellman & Hannon, 1976; Belli & Piro, 1977). Two groups have reported that the proportion of intestinal crypts that survive a given radiation dose is reduced when ADR is added to the treatment (Phillips *et al.*, 1975; Dethlefsen & Riley, 1979*b*). Dethlefsen and Riley note that this effect could be caused either by a direct, drug-induced reduction in the numbers of microcolony-forming (clonogenic) cells in each crypt or by a decrease in the radiation quasi-threshold dose ($D_q$) for each microcolony-forming cell, or both.

We demonstrate here that whole intestinal crypts can be ablated by high single doses of ADR alone, and establish a survival curve. Using such doses of ADR, a more direct estimate can then be made of the effect of the drug on the $D_q$ of irradiated microcolony-forming cells.

### MATERIALS AND METHODS

Male $B6D2F_1$(Pat) mice, aged 9–11 weeks, were used at a mean weight of 28·5 g. Animals were kept under a 12 h dark (18:00 to 06:00) 12 h light regimen and were provided with food and water *ad libitum*.

Adriamycin (Pharmitalia U.K., Barnet) was dissolved in 0·9% saline and injected i.p. as single doses. Drug concentrations were adjusted to yield injection volumes of 0·4–0·5 ml. In early experiments ADR was injected at 03:00, 09:00 or 15:00 to examine for circadian efects. No significant differences were found, and all subsequent injections were made at 15:00.

Irradiation was by a $^{137}Cs$ $\gamma$-ray unit, in which unanaesthetized mice received whole-body single doses at 15:00. In a split-dose experiment, a first dose of 10 Gy was given at 10:00 and a range of second doses 5 h later.

Intestinal response was measured by the crypt microcolony assay (Withers & Elkind, 1970). Four mice were used for each experimental point. The jejunum was removed from the animal 4 days after treatment and processed for histology. The number of regenerating crypts (microcolonies) was counted in 5 $\mu$m-thick, transverse sections of jejunum (12–30 circumferences per dose point per experiment). For each dose of agent a surviving fraction (SF) was calculated relative to untreated controls ($121 \pm 3$; all errors quoted as $\pm 2$ s.e., *i.e.*, the 95% confidence limits). Regenerating crypts often differ in size from unstimulated crypts, so a correction factor has been applied to allow for the altered probability of encountering a crypt of larger or smaller diameter in a section of given thickness (Hendry & Potten, 1974).

For comparison with the intestinal response, an ADR dose–survival curve was established for marrow "stem" cells (CFU-S) measured by the assay of Till & McCulloch (1961). Single doses of ADR were injected at 15:00. Survival of CFU-S was measured 24 h later, by injecting cell suspensions from the femurs of treated and control mice into recipients whose own marrow had been ablated by 8·5 Gy electrons. The recipients were killed 8 days later and the colonies on the spleen were counted.

RESULTS

*ADR alone: crypts*

Mice were injected with doses in the range 5–28 mg/kg body wt. Higher doses could not be used in this assay because all animals died within 2 days of injection. The proportion of crypts surviving 4 days after ADR decreased exponentially with increasing dose, after a small initial shoulder (Fig. 1). A curve was fitted to the data by a computer programme that generated Puck-type curves (Gilbert, 1969). Calculated values for the parameters reciprocal slope ($D_0$) and overall extrapolation number (N) are given in the Table.

FIG. 1.—Survival of whole crypts (●) or of femoral CFU-S (○) after single doses of ADR alone. Data points for crypts are from 4–29 repeat experiments. * indicate the approximate $LD_{50/2-8\ days}$ (crypts) and $LD_{50/20-30\ days}$ (CFU-S) for this agent. Errors as $\pm 2$ s.e.

FIG. 2.—Dose survival of crypts after various treatments. A. After 10 Gy of γ-rays (□, mean of 32 expts) followed immediately by graded single doses of ADR (■, 2–4 repeat expts); or after 12 Gy (○, 6 expts) +ADR (●, 2–4 expts). Dashed line is the first part of the curve of crypt survival for ADR alone (from Fig. 1). B. After single doses of γ-rays (dashed line, data points not included; * is the approximate $LD_{50/2-8\ days}$), and after single doses of γ-rays followed immediately by 5 mg/kg ADR (●, ○); 10 mg/kg ADR (▲, △); 15 mg/kg ADR (■, □). Errors as $\pm 2$ s.e.

TABLE.—*Mean values of parameters* ( ± 2 *s.e.*) *of the survival curves for marrow CFU-S and jejunal crypts of BDF*$_1$ *mice treated by* $\gamma$ *rays, ADR, or ADR* + $\gamma$ *rays*

| Treatment | $D_0$ | N | E | Dq |
|---|---|---|---|---|
| A. CFU-S | | | | |
| Single-dose ADR | 8·4 + 1·1 − 1·0 mg/kg | 1·30 + 0·07 − 0·07 | | |
| B. Crypts | | | | |
| Single-dose ADR | 15·9 + 15·4 − 7·8 mg/kg | 1·31 + 0·13 − 0·12 | | |
| Single-dose $\gamma$-rays | | | | |
| * | 1·41 + 0·37 Gy − 0·30 | 1228 + 28132 − 1177 | | |
| † | 1·48 + 0·36 Gy − 0·29 | 774 + 10529 − 721 | | |
| 5h-split-dose $\gamma$-rays | | | | |
| * | 1·80 + 1·09 Gy − 0·68 | 7·57 + 8·53 − 3·98 | 5·46 + 7·18 − 3·10 | 3·06 + 2·85 Gy − 1·48 |
| † | 1·48 + 0·36 Gy − 0·29 | 16·7 + 15·3 − 8·0 | 12·9 + 10·5 − 5·8 | 3·78 + 2·88 Gy − 1·63 |
| $\gamma$ + 5 mg/kg ADR | 1·68 + 1·03 Gy − 0·68 | 293 + 15436 − 291 | | |
| $\gamma$ + 10 mg/kg ADR | 2·47 + 1·06 Gy − 0·74 | 10·6 + 9·1 − 4·9 | 5·55 + 3·17 − 2·02 | 4·23 + 1·68 Gy − 1·20 |
| $\gamma$ + 15 mg/kg ADR | 3·59 + 3·78 Gy − 1·84 | 7·25 + 10·2 − 4·25 | 5·85 + 7·00 − 3·19 | 6·35 + 11·4 Gy − 4·08 |

* Independently-fitted $D_0$.

† Common $D_0$.

### *ADR alone: CFU-S*

Survival of CFU-S was measured after treatment by 4–42 mg/kg of ADR. The survival curve was fitted by an exponential function (Fig. 1). The mean $D_0$ was half that for crypts and the mean value of N was the same as that for the *whole* crypts (Table).

### *Radiation alone: crypts*

The survival curve for single doses of $\gamma$-rays had a large threshold of $\sim$8 Gy (Fig. 2B). The independently fitted curves for single and split doses of radiation had different mean $D_0$s (Table) so that values of $D_2$–$D_1$ varied somewhat with level of survival, being 3·3 Gy at SF $10^{-1}$ and 4·35 Gy at $10^{-2}$.

### *Radiation + ADR, crypts*

1. Single doses of 1–11 mg/kg ADR were injected immediately (within 1 min) after the mice were irradiated with 10 or 12 Gy of $\gamma$-rays. Crypt survival decreased with increasing ADR dose, with little evidence of a marked shoulder on either curve (Fig. 2A).

2. Single doses of 5, 10 or 15 mg/kg of ADR were injected immediately after single doses of $\gamma$-rays in the range 2–15·25 Gy. All 3 curves fell to the left of that for single doses of radiation alone (Fig. 2B) but retained a large threshold or shoulder region. The mean $D_0$ tended to increase with the dose of drug in the combination (Table).

### DISCUSSION

A survival curve has been established for unstimulated jejunal crypts of BDF$_1$ mice treated by ADR alone. The curve differed from that for marrow CFU-S of these mice in having twice the shoulder (4 and 2 mg/kg respectively) and twice the $D_0$ (15·9 and 8·4 respectively). Hellman & Hannon (1976) measured the survival of resting femoral CFU-S from mice treated

3 h earlier by an i.v. injection of ADR. The survival curve had a very small shoulder (1 mg/kg) and a $D_0$ of ~26 mg/kg (estimated from a graph). Of other normal tissues in mice, unstimulated telogen hair coat was found to be very resistant to the action of ADR, as measured in terms of hair loss, but stimulated-telogen or anagen hair had a $D_0$ of ~8–10 mg/kg (estimated from graphs in Griem *et al.*, 1979). The stem cells of the testes are highly sensitive; Lu & Meistrich (1979) obtained a $D_0$ of 1·33 mg/kg of ADR, with a threshold dose of 5 mg/kg. It appears then, that in relation to other normal tissues, the microcolony-forming cells of the unperturbed jejunum are moderately sensitive to the cytotoxic action of ADR.

A feature of the crypt survival curve for ADR was that the overall extrapolation number (N) was much lower than that for single doses of $\gamma$-rays (1·3(+0·13/−0·12) and 1233(+28132/−1177) respectively). The shoulder on curves of crypt survival, as reflected by N, is generally held to be the product of 2 components: the *number* of microcolony-forming cells per crypt (A) and the capacity of *each* microcolony-forming cell to accumulate sublethal damage (SLD). This shoulder on the survival curve for individual microcolony-forming cells, measured by the parameter $D_q$, contributes to N in the form of the single-cell extrapolation number E. The value of E and hence of A (=N/E) can be estimated from curves for single doses of agent and 2 doses separated by an interval sufficient to allow full repair of SLD. This calculation of A assumes a common $D_0$ for the 2 curves, an assumption that is not wholly supported by our results (the independently-calculated $D_0$s for single and 5h-split doses of $\gamma$-rays were 1·41 and 1·80 Gy respectively; $P<0{\cdot}025$). Fitting a common $D_0$ to the two sets of data decreased the value of N for single doses and raised the extrapolation number of the split-dose curve (Table). Adopting the common-fit calculations, Poisson statistics indicate that after a $D_1$ of 10 Gy, each surviving crypt should contain on average 1·3 surviving microcolony-forming cells. Allowing for this multiplicity, the value of the intercept on the ordinate of the split-dose curve (E) was 12·9(+10·5/−5·8). For a single-dose N of 774 the average number of microcolony-forming cells would therefore be 60(+345/−51). The errors associated with such estimates are very large, but to our knowledge all results obtained by this method for the X- or $\gamma$-irradiated small intestine imply the existence of several microcolony-forming cells per crypt (15–150; summarized in Yau & Cairnie, 1979).

The results for crypts ablated by ADR alone are quite different. It is assumed that there is no accumulation of SLD in ADR-treated cells (note the absence of a shoulder on the curves for low doses of ADR, Fig. 2A). The overall extrapolation number should therefore represent also the number of clonogenic cells per crypt, *i.e.* 1 or 2 (N=1·31(+0·13/−0·12)). This interpretation assumes a uniform sensitivity to the agent of all potentially clonogenic cells. It is possible that the crypt contains large numbers of clonogenic cells the majority of which are sensitive to ADR, so that the observed survival curve beginning at 6 mg/kg is that of a small resistant sub-population. If the crypt contains 60 microcolony-forming cells and 6 mg/kg is the dose that reduces survival to the "resistant" component, the "sensitive" sub-population would have a $D_0$ of 1·5–2·0 mg/kg. This is close to the value for the highly sensitive spermatogenic stem cells (Lu & Meistrich, 1979). This hypothetical sub-population cannot include the radiation-resistant microcolony-forming cells that survive doses of 10–12 Gy. The slopes of the ADR survival curves for these cells were not markedly less than for high doses of ADR (11·4 and 11·8 mg/kg respectively; Fig. 2A). The argument for a non-uniform population also requires that most microcolony-forming cells are very sensitive to a variety of drugs; low extrapolation numbers have also been found on curves of crypt survival for the alkylating agents mechlorethamine hydrochloride and isopropyl methane sulphonate (Moore, 1979) and the antibiotic bleomycin (Moore, unpublished).

It may be queried whether each crypt contains a fixed high number of microcolony-forming cells, constituting a discrete functional compartment, but whose number is not necessarily derivable directly from the N on survival curves for whole crypts, or whether the value of N *does* permit an estimate of A, but the effective target population varies in size for different agents.

It is clear that ADR destroys epithelial cells of the intestinal mucosa (Dethlefsen & Riley, 1979*a*) and we have now demonstrated that clonogenic cells are killed. We could not show that ADR enhances the effect of simultaneously-delivered radiation by reducing the $D_q$ for microcolony-forming cells (Table). Calculated values for the single-cell extrapolation number E were the same for radiation alone and for radiation plus ADR. The $D_0$ of the radiation survival curve tended to increase with increasing dose of ADR, so that the calculated mean $D_q$ rose also. These results contrast with those for V79 Chinese hamster cells *in vitro*, for which the $D_q$ decreased when ADR was given either simultaneously with or 2 h after radiation (Belli & Piro, 1977). Dethlefsen & Riley 1979*b*) found an increase in $D_0$ when ADR preceded X-rays by 7 days, but not when the two were separated by 2 h.

We conclude that the shift to the left observed in survival curves for intestinal crypts treated by ADR$^+$ radiation is caused very largely by direct drug cytotoxicity, and not by enhancement of the radiation response of microcolony-forming cells.

### REFERENCES

Belli, J. A. & Piro, A. J. (1977) The interaction between radiation and adriamycin damage in mammalian cells. *Cancer Res.*, **37**, 1624.

Dethlefsen, L. A. & Riley, R. M. (1979*a*) The effects of adriamycin on murine duodenal crypt cell proliferation. *Int. J. Radiat. Oncol. Biol. Phys.*, **5**, 501.

Dethlefsen, L. A. & Riley, R. M. (1979*b*) The effects of adriamycin and X-irradiation on the murine duodenum. *Int. J. Radiat. Oncol. Biol. Phys.*, **5**, 507.

Gilbert, C. W. (1969) Computer programmes for fitting Puck and probit survival curves. *Int. J. Radiat. Biol.*, **16**, 323.

Griem, M. L., Dimitrievich, G. S. & Lee, R. M. (1979) The effects of X-irradiation and adriamycin on proliferating and non-proliferating hair coat of the mouse. *Int. J. Radiat. Oncol. Biol. Phys.*, **5**, 1261.

Hellman, S. & Hannon, E. (1976) Effects of adriamycin on the radiation response of murine haematopoietic stem cells. *Radiat. Res.*, **67**, 162.

Hendry, J. H. & Potten, C. S. (1974) Cryptogenic cells and proliferative cells in intestinal epithelium. *Int. J Radiat. Biol.*, **25**, 583.

Lu, C. C. & Meistrich, M. L. (1979) Cytotoxic effects of chemotherapeutic drugs on mouse testis cells. *Cancer Res.*, **39**, 3575.

Moore, J. V. (1979) Ablation of murine jejunal crypts by alkylating agents. *Br. J. Cancer*, **39**, 175.

Phillips, T. L., Wharam, M. D. & Margolis, L. W. (1975) Modification of radiation injury to normal tissues by chemotherapeutic agents. *Cancer*, **35**, 1678.

Till, J. E. & McCulloch, E. A. (1961) A direct measurement of the radiation sensitivity of normal mouse bone marrow cells. *Radiat. Res.*, **14**, 213.

Withers, H. R. & Elkind, M. M. (1970) Microcolony survival assay for cells of mouse intestinal mucosa exposed to radiation. *Int. J. Radiat. Biol.*, **17**, 261.

Yau, H. C. & Cairnie, A. B. (1979) Cell-survival characteristics of intestinal stem cells and crypts of gamma-irradiated mice. *Radiat. Res.*, **80**, 92.